# Precancerous cervical lesion and associated factors among HIV-infected women in Ethiopia: systematic review and meta- analysis

**DOI:** 10.1186/s12885-024-12462-9

**Published:** 2024-06-03

**Authors:** Yeshiwas Ayale Ferede, Worku Chekol Tassew, Agerie Mengistie Zeleke

**Affiliations:** 1 Department of Reproductive Health, Teda Health Science College, Gondar, Ethiopia; 2 Department of Medical Nursing, Teda Health Science College, Gondar, Ethiopia; 3 Department of Clinical Midwifery, Teda Health Science College, Gondar, Ethiopia

## Abstract

**Background:**

Despite several preventative and control measures Ethiopia continues to see an increase in cervical cancer. Comprehensive evidence is very important to suggest ministry of health. Therefore, the aim of this study is to estimate the pooled violence of Precancerous Cervical Lesion and to identify associated factors among women living with HIV AIDS in Ethiopia.

**Methods:**

From February 15, 2024 to March 17, 2024, systematic and methodical search of the literature was conducted using electronic databases such as PubMed, HINARI, Global Health, Scopus, EMBASE, Web of Science, African Journal online (AJOL), and Google Scholar. Quality appraisal was assessed based on Joanna Briggs Institute (JBI) critical appraisal checklist for analytical cross-sectional study using 9 criteria. The Cochrane Q and I2 test statistics were used to verify the heterogeneity of the studies. Using a fixed effect model, the pooled estimate prevalence of precancerous cervical lesion among women living with HIV was calculated.

**Results:**

After reviewing 9,470 studies, 9 studies involving 2,910 women with HIV were included. The pooled estimate of precancerous cervical cancer among women living with HIV in Ethiopia was 15.34% (95% CI: 8.97, 21.72). Having history of sexual infection (POR = 3.12; 95% CI: 1.38, 7.05), having multiple sexual partner (POR = 3.14; 95% CI: 2.29, 4.30), and parity greater than two (POR = 4.97; 95% CI: 3.17, 7.78) were identified factors associated with precancerous cervical lesion.

**Conclusion:**

This study found that about one-six of HIV-positive women developed precancerous cervical lesion. According to this study, there was a substantial correlation between precancerous cervical lesion among HIV-positive women and having history of sexually transmitted infection, having multiple sexual partners, and being multipara. In order to reduce precancerous cervical lesion, FMOH, policy makers, and interested parties should pay particular attention to this issue.

**Supplementary Information:**

The online version contains supplementary material available at 10.1186/s12885-024-12462-9.

Keywords prevalence, cervical lesion, associated factors, HIV –positive women, Ethiopia, systematic review and meta-analysis.

## Background

Cervical cancer is the fourth most common cancer in women globally with around 660 000 new cases and around 350 000 deaths in 2022 [1]. Nonetheless, approximately 90% of cervical cancer deaths occur in poorer nations, making it the fourth most common cause of cancer-related deaths among women worldwide. Approximately 22% of cervical cancer cases worldwide are in sub-Saharan Africa [2–4]. By 2030, 443,000 deaths are predicted, a 67% increase in the current number of deaths [5]. An estimated 5000 people in Ethiopia pass away from cervical cancer each year, along with 7000 new cases [6].

A disproportionate lack of screening is the main cause of the high incidence of cervical cancer in sub-Saharan Africa, some regions of Latin America and the Caribbean, and other medically underserved groups [4]. Sexually transmitted infections such as human papillomavirus (HPV) are linked to a higher prevalence of HIV infection, particularly in sub-Saharan Africa [7–10]. Women who are HIV-positive face an increased risk of acquiring human papillomavirus (HPV), especially when their CD4 + cell count is low. The use of antiretroviral therapy (ART) has been shown to decrease the likelihood of HPV acquisition, increase the body’s ability to clear the virus, and decrease the progression to precancerous stages. This effect is likely due to the restoration of the immune system’s functionality [11]. In Ethiopia, HPV-related risk factors for cervical cancer include cultural factors, poverty, coinfection, and lack of awareness [12]. Moreover, Ethiopian women usually seek cancer care at a late stage of the disease, when therapy is probably ineffective, and there is no uniform strategy or methodology for cervical cancer screening; instead, it is patchy or inconsistent [6].

Cervical cancer-related morbidities and deaths are considerably reduced when high-risk and vulnerable groups are screened for asymptomatic precancerous cervical lesions [13]. In Ethiopia, cervical cancer is a serious concern, especially since 99% of HIV + women undergo a Visual Inspection Acetic acid(VIA) test [14]. To fulfill the 2020 aim of screening at least 80% of women aged 30–49 years, a precancerous cervical lesion (PCCL) screening program has been established [15]. Despite several preventative and control measures, there is a continuing increase in the incidence of Ethiopia among patients with cervical cancer. The 2016 Ethiopian Demographic Health Survey (EDHS) revealed that 1.2% of women between the ages of 15 and 49 had HIV/AIDS [16]. A total of 534,000 Ethiopian women who were 15 years of age or older with HIV were included. Due to their tenfold increased risk of precancerous lesions and increased likelihood of developing invasive cervical cancer compared to those of uninfected women, these women are among the most susceptible to the disease [10, 14]. According to WHO guidelines, all sexually active women between the ages of 30 and 49 years should undergo cervical cancer screening at least once every five years. However, HIV-positive women of any age should undergo screening every three years [2]. Ethiopia embraced the WHO recommendation in 2015, advising HIV-positive women to start screening as soon as they receive their diagnosis, regardless of age, and to rescreen every five years [14]. The government of Ethiopia has increased its focus on early detection programs. Various stakeholders, including professionals, academics, the media, and development partners, have launched several advocacy campaigns to combat cervical cancer [14].

In this study, the literature on precancerous cervical lesions among women living with HIV AIDS in Ethiopia was reviewed. However, studies have shown differences in precancerous cervical lesions and associated factors, and to the knowledge of the investigator concerned, the literature has not been systematically examined. Therefore, this systematic review and meta-analysis aimed to estimate the pooled incidence of cancer involving cervical lesions and to identify associated factors among women living with HIV-AIDS in Ethiopia. The findings of this meta-analysis will help policy makers and other stakeholders effectively implement the prevention and control of precancerous cervical lesions.

## Materials and methods

This systematic review and meta-analysis was performed based on the Preferred Reporting Items for Systematic Review and Meta-Analysis (PRISMA) guidelines [17].

### Search strategy

A thorough and methodical review of the literature was conducted across various electronic databases, such as PubMed, HINARI, Global Health, Scopus, EMBASE, Web of Science, African Journal Online (AJOL), and Google Scholar, spanning from February 15, 2024, to March 17, 2024. Additionally, manual searches were performed across different repositories to identify unpublished studies and gray literature. The search utilized a combination of keywords and Medical Subject Headings (MeSH) terms. Specifically, the search strategy included the following terms: “precancerous” (MeSH), “cervical lesion”, “Cervical Intraepithelial Neoplasia” (MeSH), “factors”, “Determinants” (MeSH), “HIV-positive”, “HIV Seropositivity” (MeSH), “women” (MeSH), and “Ethiopia” (MeSH). Boolean operators (AND, OR), truncation, and appropriate application of MeSH terms were employed in the systematic search. The focus was on identifying epidemiological studies concerning precancerous cervical lesions and their associated factors among HIV-positive women in Ethiopia.

### Inclusion and exclusion criteria

Original research studies reporting on precancerous cervical lesions and/or associated factors among HIV-positive women in Ethiopia were included in the study. Both published and unpublished articles written only in the English language were considered for inclusion. All publications reported up to March 17, 2024, were considered. Studies that did not clearly report precancerous cervical lesions among HIV-positive women in Ethiopia were excluded. In addition, articles without full texts, abstracts, editorial reports, letters, reviews, and commentaries were excluded from the study.

### Data extraction

Following the screening of titles, abstracts, and full texts of each selected original study, data extraction was carried out using a standardized tool adapted from the Joanna Briggs Institute (JBI). Two independent reviewers (YAF & AMZ) performed the data extraction process and thoroughly reviewed all included articles. Any disagreements between the reviewers were resolved through discussion. Various study characteristics, including the first author’s name, study region, publication year, study design, participants, sampling technique, and sample size, were extracted. Additionally, the prevalence of precancerous cervical lesions and associated risk factors, along with their corresponding 95% confidence intervals, were also extracted.

### Risk of bias (quality) assessment

The assessment of study quality was conducted using the Joanna Briggs Institute (JBI) critical appraisal checklist for analytical cross-sectional studies, which comprises nine criteria [18]. These criteria included [1] appropriateness of the sample frame for addressing the target population [2], appropriateness of participant sampling [3], adequacy of sample size [4], detailed description of study subjects and setting [5], thoroughness of data analysis covering the identified sample [6], utilization of valid methods for condition identification [7], measurement of the condition in a standardized and reliable manner for all participants [8], appropriate statistical analysis, and [9] adequacy of response rate. Each criterion was scored as 0 for ‘not reported or not appropriate’ and 1 for ‘yes’. The scores across these items were then aggregated to obtain a total quality score ranging from 0 to 9. Subsequently, studies were categorized as low, medium, or high quality based on the total points awarded: 0–4 for low quality, 5–7 for medium quality, and 7–9 for high quality.

### Outcome measurement

This review focused on two main outcomes. The primary outcome addressed in this systematic review and meta-analysis pertains to precancerous cervical lesions. These lesions are characterized by dense acetowite areas with well-defined margins observed in proximity to the transformation zone or if the entire cervix or cervical growth turns white during VIA. Classification as negative, positive, or suspicious for invasive cervical cancer (ICC) followed the guidelines outlined in the International Agency for Research on Cancer (IARC) training manual [19].

The second outcome variable of interest in the study was the identification of factors associated with precancerous cervical lesions among HIV-positive women in Ethiopia, assessed in terms of the odds ratio (AOR). The odds ratio for each identified factor was calculated based on the binary outcome data reported in each primary study.

### Data synthesis and analysis

The data extraction process involved utilizing a Microsoft Excel spreadsheet, followed by importing the data into STATA version 11 for subsequent analysis. To describe and summarize the primary studies, tables, figures, and forest plots were used. The pooled estimate of precancerous cervical lesions was calculated using a fixed-effects model, along with a 95% confidence interval (CI). For the assessment of factors associated with precancerous cervical lesions among HIV-positive women, associations were estimated using odds ratios with corresponding 95% CIs. A fixed-effects model was employed during the meta-analysis due to the demonstrated homogeneity among the included studies. The assessment of heterogeneity among the included studies was conducted using Cochran’s Q statistic and I² statistics. Additionally, visual inspection of asymmetry in funnel plots and Egger regression tests were performed, with a p value of less than 0.05 serving as the cutoff point to indicate the presence of publication bias.

## Results

### Study selection

**Fig. 1:**
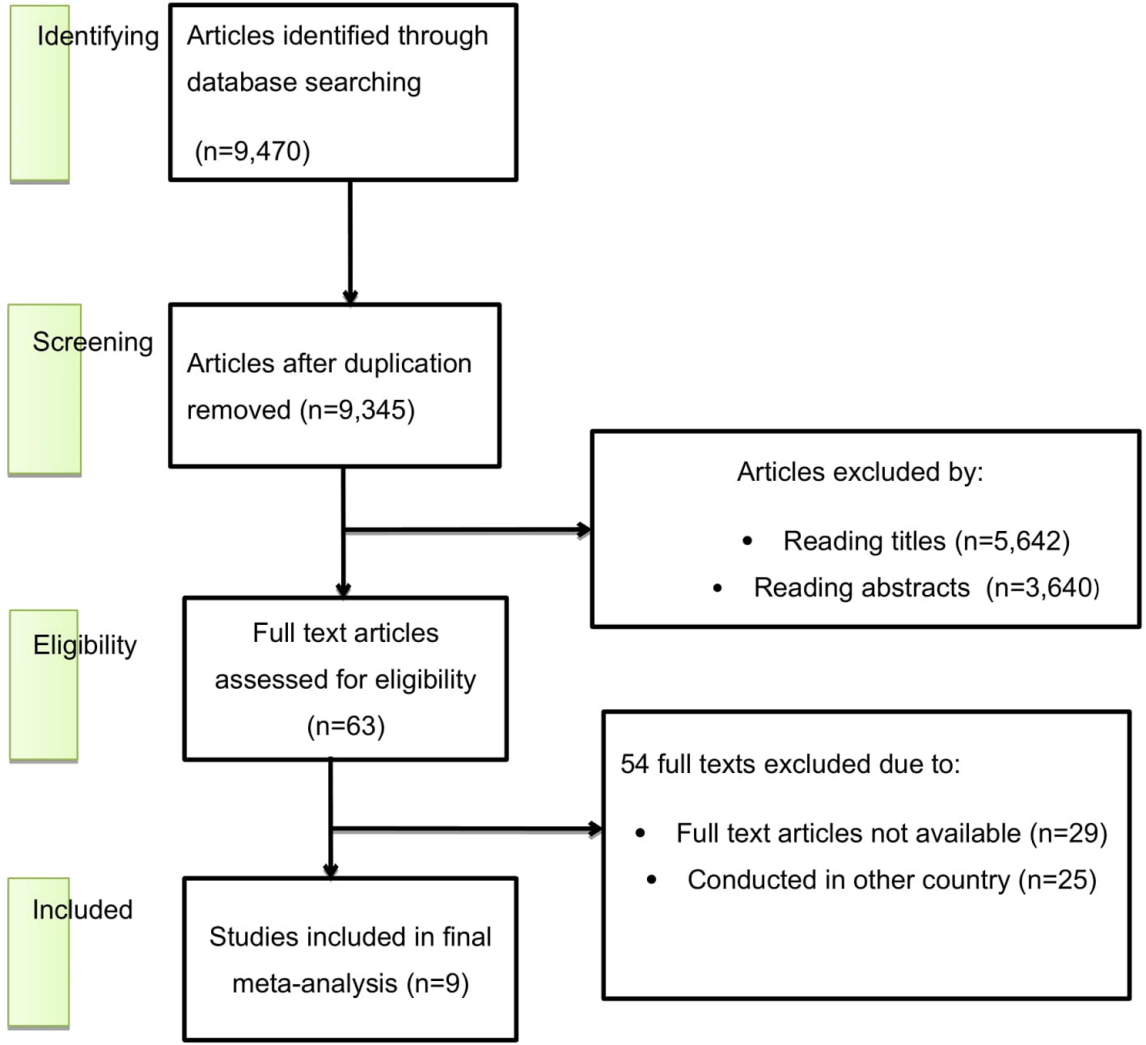
Flow chart of study selection for systematic review and meta-analysis of precancerous cervical lesion and associated factors among HIV-positive women in Ethiopia, 2024

Out of a total of 9,470 articles retrieved regarding precancerous cervical lesions and/or associated factors among women living with HIV/AIDS in Ethiopia, 125 duplicates were identified and removed. Subsequently, 9,282 articles were excluded after their titles and abstracts were evaluated based on predetermined criteria. After the evaluation of the full texts of the remaining 63 articles against the qualifying criteria, 54 studies were further excluded, primarily because they were published outside of Ethiopia or because of differences in the study population. Consequently, nine studies met the inclusion criteria and were included in the final meta-analysis (Fig 1).

### Characteristics of the included studies

Of the included studies, eight were institutional-based and cross-sectional, and the remaining study was a case‒control study. All studies that were included in this evaluation were published between 2013 and 2023. Among the papers included in this review, four involved systematic sampling, one involved simple random sampling, one involved consecutive sampling, and the remaining three studies did not report the sampling technique used. There were 2,910 participants from an estimated 2,969 HIV-positive women, with an estimated sample size ranging from 129 [20] to 458 [21]. According to the included research, the prevalence of precancerous cervical lesions ranged from 7.5% [15] to 22.1% [21]. The Amhara area was the focus of five of the studies included in this review [9, 20, 22–24]; two investigations were conducted in the Southern Nations, Nationalities, and People’s Region (SNNPR) [21, 25], and the remaining two were conducted in the Addis Ababa and Oromia Regions[15, 26] Table 1.

**Table 1:** Descriptive summary of primary studies included in the meta-analysis of precancerous cervical cancer and associated factors among women living with HIV in Ethiopia, 2024

id	Author	Pub year	Region	study design	Study population	Sample size	Prevalence (%)	Response rate (%)
1	Belayneh, et al. [13]	2019	Amhara	institutional based cross-sectional	HIV -positive women	291	9.9	97.6
2	Zelalem et al [17]	2022	Addiss Ababa	institutional based cross-sectional	HIV -positive women	267	7.5	100
3	Mulugeta, Yaze [25]	2022	Amhara	institutionalbased comparative cross-sectional	HIV -positive women	366	16.6	91.2
4	Kiros et al. [23]	2022	Amhara	institutionalbased comparative cross-sectional	HIV -positive women	129	9.3	100
5	LEMMA ET AL [29]	2023	Oromia	institutional based cross-sectional	HIV -positive women	257	16	100
6	Abel Gedefaw [24]	2013	southern	institutional based cross-sectional	HIV -positive women	458	22.1	97.6
7	Lemu et al [28]	2021	southern	institutional based cross-sectional	HIV -positive women	454	18.7	97.8
8	Mulugeta Dile [26]	2019	Amhara	institutional based cross-sectional	HIV -positive women	435	20.2	100
9	Dessie et al [27]	2023	Amhara	case-control	HIV -positive women	312	9.9	100

### Meta-analysis

#### Risk of bias assessment for the included studies

The critical evaluation checklist developed by the Joanna Briggs Institute (JBI) and adjusted for cross-sectional studies. The quality evaluation summary showed two-third (*n* = 6, 66.66%) of the included studies had high quality while the remaining one-third (*n* = 3, 33.33%) of studies had medium quality.

#### Precancerous cervical lesion

**Fig. 2:**
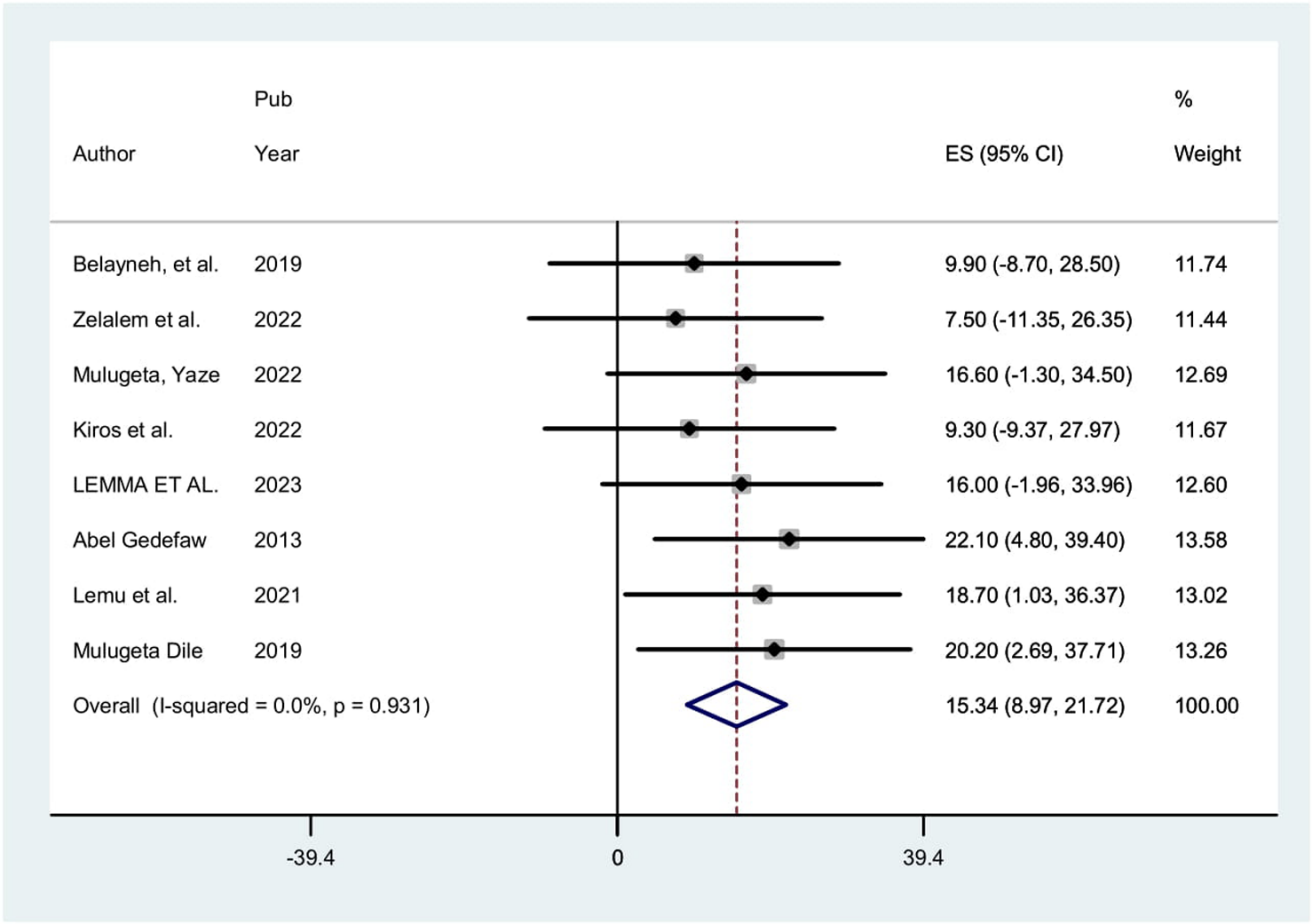
Forest plot of the pooled prevalence of precancerous cervical lesion among HIV-positive women in Ethiopia, 2024

In this study, the pooled estimate of Precancerous cervical lesion among HIV-positive women was 15.34% (95% CI: 8.97, 21.72). In estimating the pooled prevalence of Precancerous cervical lesion among HIV-positive women homogeneity through the included studies was exhibited (I2 = 0.00%; p *=* 0.931). Therefore, a fixed effects model was used in the meta-analysis to calculate the pooled prevalence of Precancerous cervical lesion (Fig. 2).

##### Publication bias

**Fig. 3:**
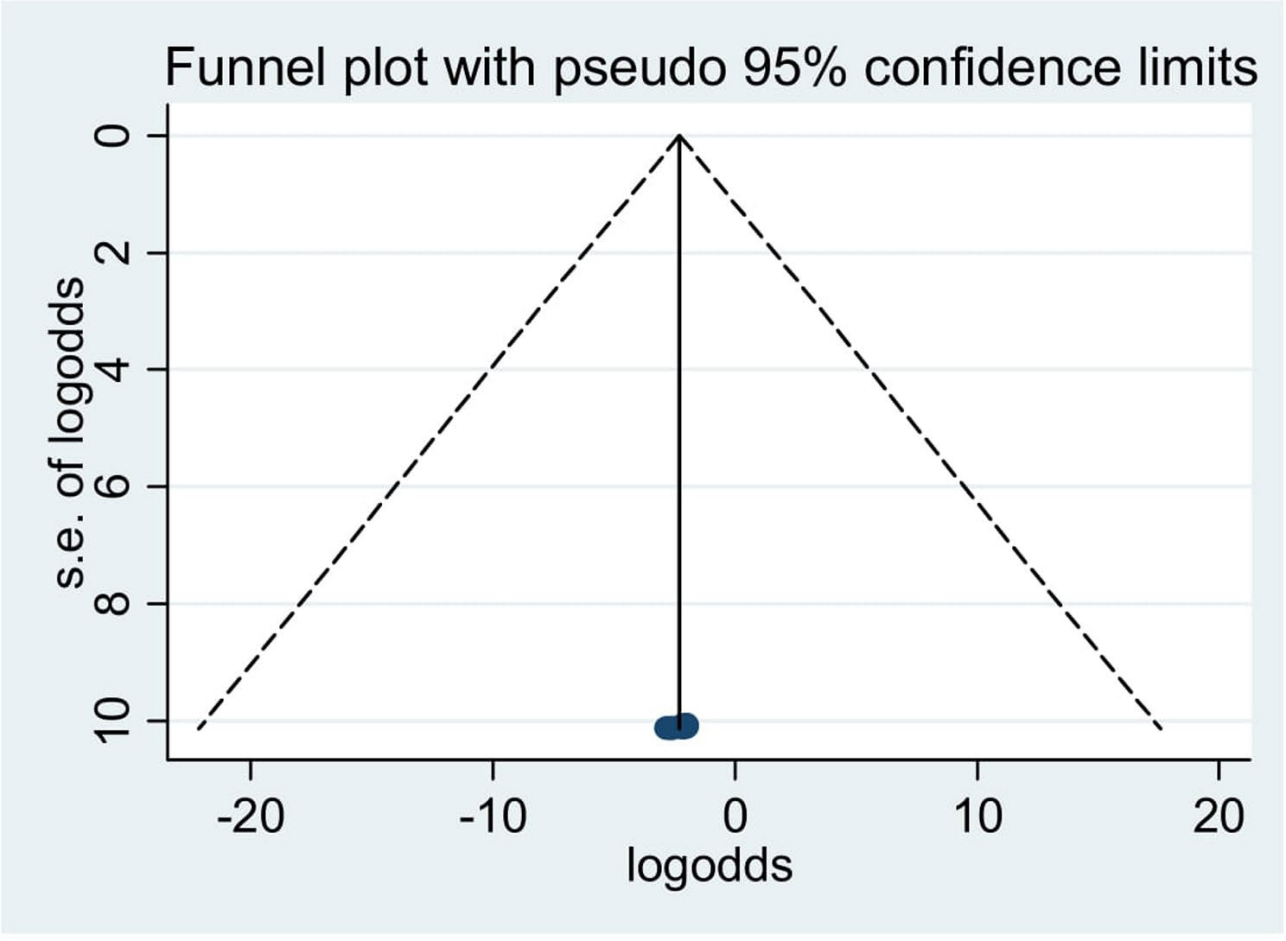
Showing publication bias using funnel plot, 2024

To determine if publication bias existed, a visual examination of the asymmetry in funnel plots and Egger regression tests were used. As a consequence, the results of Egger’s tests and funnel plots indicated that publication bias existed in the included papers. Egger’s test revealed the existence of publication bias with a statistically significant result (*p* = 0.000). Additionally, an examination of the funnel plots visually revealed an uneven distribution of the study (Fig. 3).

##### Trim and fill analysis

Because there is publication bias, Trim and fill analysis was done. Trim and fill analysis findings showed that one study was removed.

#### Associated factors

In this study, certain factors associated to precancerous cervical lesion were statistically pooled, but some weren’t since the independent variables were not evenly classified or grouped about the outcome, which is precancerous cervical lesion.

**Fig. 4:**
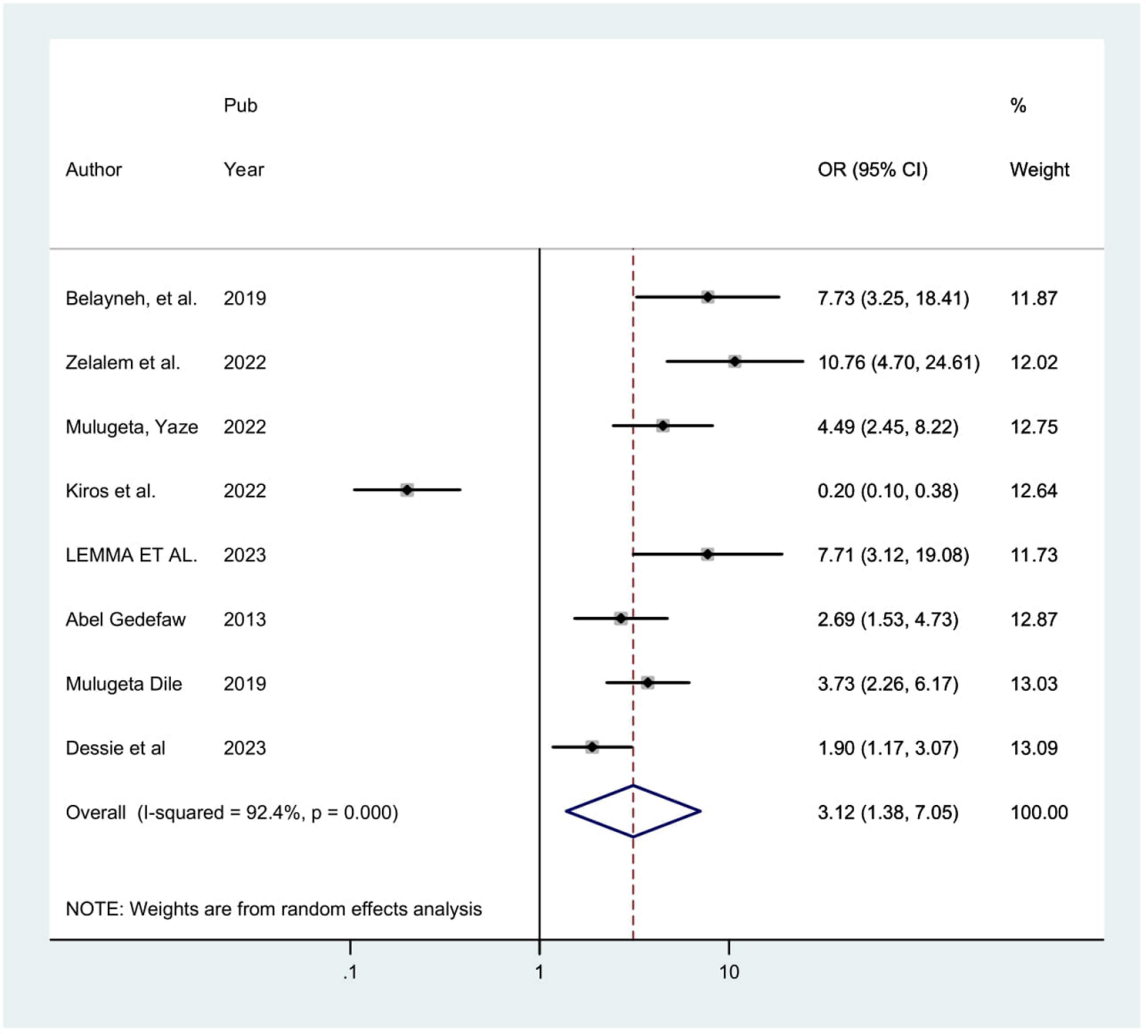
Showing the forest plot of the association between sexual transmitted infection and precancerous cervical lesion in Ethiopia, 2024

Eight studies indicated that having history sexually transmitted infection has a substantial correlation with precancerous cervical lesion. The odds of precancerous cervical lesion were 3.12 times (POR = 3.12; 95% CI: 1.38, 7.05) higher among Womens who had history sexually transmitted infection when compared with those who hadn’t history sexually transmitted infection. This meta-analysis revealed considerable heterogeneity among the included studies (I2 = 92.4%, *P* = 0.000). Thus, an analysis using a random effect model was employed (Fig. 4).

**Fig. 5:**
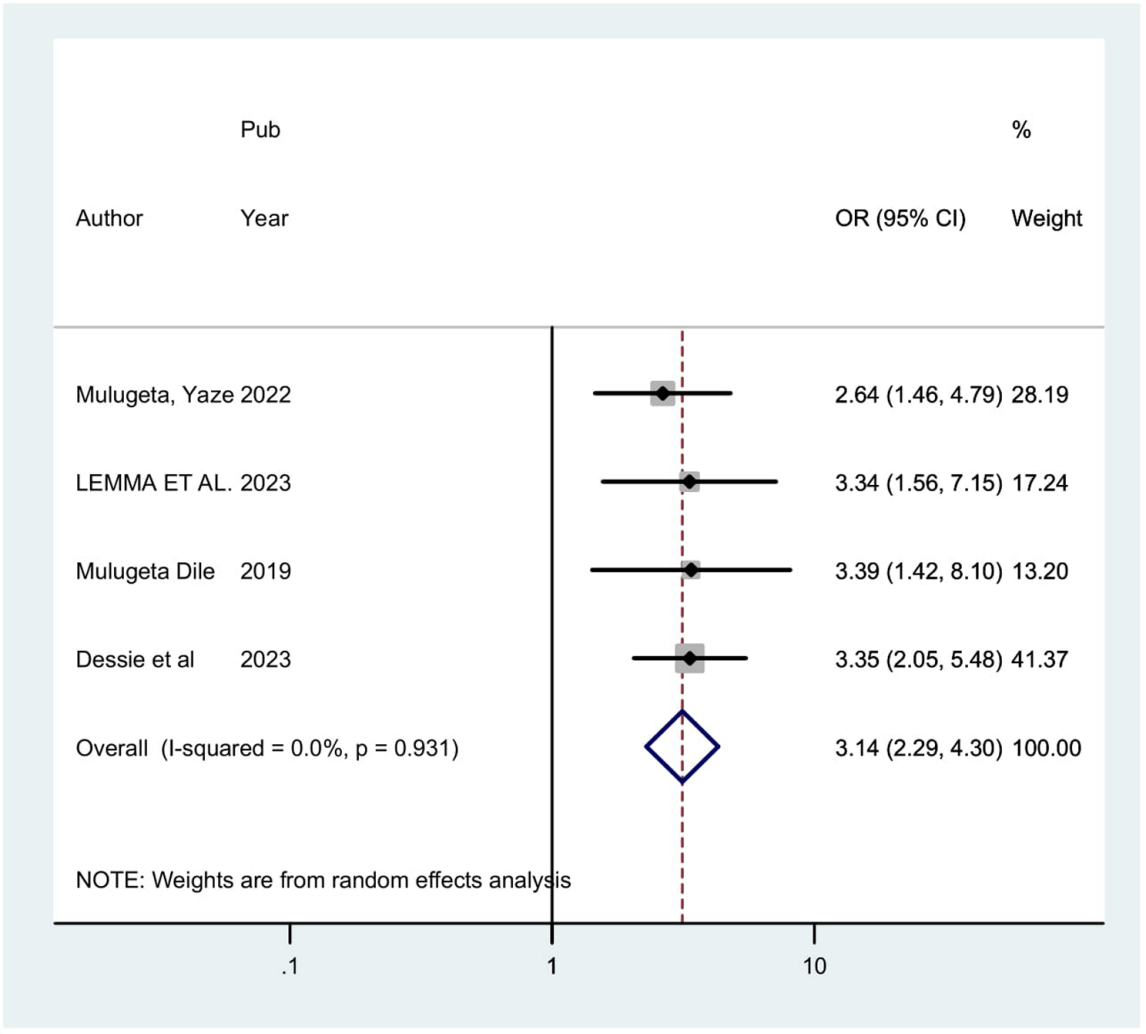
Showing the forest plot of the association life time sexual partner and precancerous cervical lesion in Ethiopia, 2024

Four studies indicated that sexual partner has a substantial correlation with precancerous cervical lesion. The odds of precancerous cervical lesion were 3.14 times (POR = 3.14; 95% CI: 2.29, 4.30) higher among HIV positive women who had multiple sexual partner when compared with those hadn’t multiple sexual partner. The homogeneity of the included studies in this analysis was noted (I2 = 0.0%, *P* = 0.931). Thus, a fixed effect model analysis was applied (Fig. 5).

**Fig. 6:**
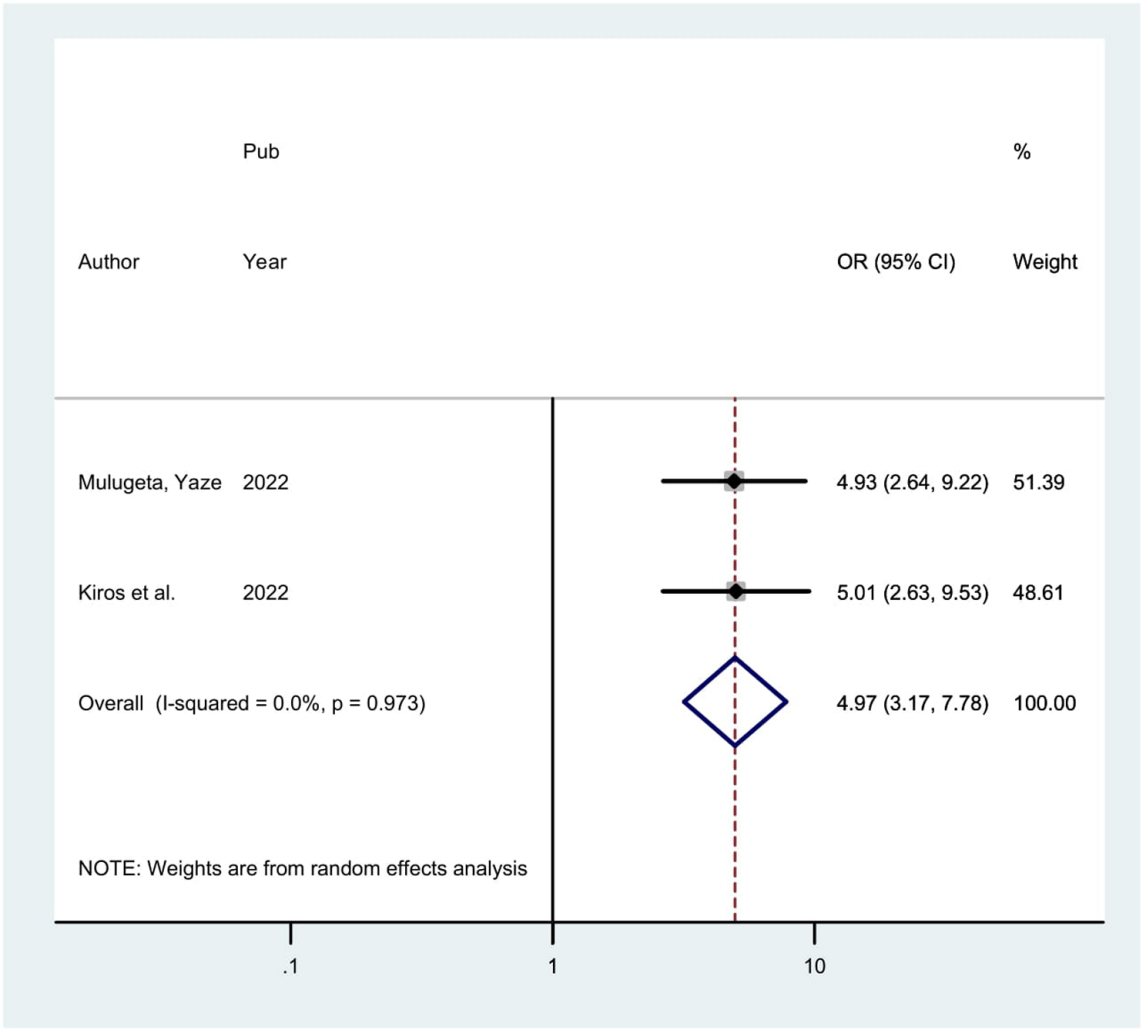
Showing the forest plot of the association between parity and precancerous cervical lesion in Ethiopia, 2024

Furthermore: Two studies indicated that parity has a substantial correlation with precancerous cervical lesion. The odds of precancerous cervical lesion were 4.97 times (POR = 4.97; 95% CI: 3.17, 7.78) higher among women whose parity greater than two when compared with those whose parity less than or equal two. Homogeneity was found in the analysis of the included studies (I2 = 0.0%, *P* = 0.973). Thus, an analysis using a fixed effect model was employed (Fig. 6).

## Discussion

The purpose of this meta-analysis was to estimate the prevalence of precancerous cervical lesion nationwide and its associated factors. This meta-analysis, to the best of our knowledge, is the first of its type to calculate the pooled prevalence of precancerous cervical lesion and associated factors among women living with HIV in Ethiopian. According to this analysis, there is a broad range of precancerous cervical lesion among HIV-positive women from 7.5 to 22.1%. In Ethiopia, the pooled prevalence of precancerous cervical lesion among women living with HIV was 15.34% (95% CI: 8.97, 21.72). This finding strongly showed that being HIV-positive means at risk for developing a precancerous cervical lesion. The current study’s findings on precancerous cervical lesion among women living with HIV are in line with other Studies recently conducted in Côte d’Ivoire(11%) [27], however higher than the study conducted in Nigeria found that the prevalence of precancerous cervical cancer lesion to be 6% [28]. On the other hand, this study was lower than the studies conducted in Kenya (26.7%), Rwanda (24.3%), Uganda (73%), and Zambia (76%), in Sub-Saharan Africa.

25.6% [29], and South Africa (66.3%) [30–34], .

According to this study, women who had higher parity had a higher risk of precancerous cervical lesions. This finding is in line with the findings of earlier research carried out in Tanzania(33 and Côte d’Ivoire [35]. This is because frequent vaginal deliveries put the woman at risk of HPV infection by tearing of the vaginal wall, which allows for cross-contamination. On the other hand, study done in Rwandan found that the likelihood of getting a cervical cancer lesion declined as party number increased [36].

According to this study, women with a history of sexually transmitted infections (STIs) were 3.12 times more likely to develop a precancerous cervical lesion than women without a history of STIs. This finding is consistent with research from Uganda, which found that women without a history of STIs had a 76% lower risk of developing precancerous cervical lesions [37]. Most of the available data pointed to a viral infection as the primary cause of cervical cancer and suggested that the virus is transmitted through sex. For example, there is evidence linking the severity of aberrant cervical cytology and HPV infection to chlamydia trachomatis infection.

Furthermore, the current study discovered that women who had several sexual partners throughout the course of their lifetimes were 2.53 times more likely than women who had only one partner to acquire precancerous cervical lesions. This is because unprotected sexual contact is one method that HPV may spread, which could explain why women who have several sexual partners may be at a higher risk of contracting the virus [38]. Therefore, health practitioners who routinely care for HIV-positive women should prescribe the VIA test in addition to advising women to limit the number of sexual partners to lower the prevalence of PCCL.

This study showed that women with higher parity were more likely to have precancerous cervical lesion compared. This finding is consistent with the results from the previous studies conducted in Tanzania [39] and Côte d’Ivoire [35] This is due to repeated vaginal delivery might be exposed to human papillo virus strains (HPVs) infection due to vaginal wall laceration which is conducive for cross contamination. However, in contrast, another study done in Rwanda indicated the risk of developing any cervical cancerous lesion decreased with increasing party [36].

Despite being the first systematic review and meta-analysis on precancerous cervical lesion among Ethiopian women living with HIV, this study has many shortcomings. This meta-analysis includes full-text articles that were published only in English. The pooled odds ratio for all variables associated to precancerous cervical lesion among women living with HIV was not examined as the included studies had varying definitions of the variables. The facility-based nature of the cross-sectional research seen in all of the included articles that were considered may restrict the generalizability of the findings. Additionally, this analysis only included studies from four regions, which may affect the generalizability of the findings at the national level. Furthermore, the presence of publication bias is also another limitation of this meta-analysis.

## Conclusion

This study found that about one-six of HIV-positive women developed precancerous cervical lesion. According to this study, there was a substantial correlation between precancerous cervical lesion among HIV-positive women and having history of sexually transmitted infection, having multiple sexual partners, and being multipara. In order to reduce precancerous cervical lesion, FMOH, policy makers, and interested parties should pay particular attention to this issue.

### Electronic supplementary material

Below is the link to the electronic supplementary material.

Supplementary Material 1

## Data Availability

All relevant data generated and analyzed is included in this article.

## Abbreviations

POR: Pooled odd ratio
AOR: Adjusted odd ratio
CI: Confidence intervals
PRISMA: Preferred Reporting Items for Systematic review and Meta-analysis
SNNPR: South Nation and Nationality Peoples Regional
FMOH: Federal, Ministry, of Health
PCCL: Precancerous cervical lesion
IARC: International Agency for Research on Cancer
VIA: Visual Inspection Acetic acid
